# Uterine prolapse in pregnancy: a case report and literature review

**DOI:** 10.11604/pamj.2024.49.101.43712

**Published:** 2024-12-02

**Authors:** Hanane Houmaid, Karam Harou, Bouchra Fakhir, Lahcen Boukhanni, Ahlam Bassir, Abderrahim Aboulfalah, Hamid Asmouki, Abderraouf Soummani

**Affiliations:** 1 Department of Obstetrics and Gynecology, Mohammed VI University Hospital Center, Faculty of Medicine and Pharmacy, Marrakech, Morocco

**Keywords:** Uterine prolapse, pregnancy, vaginal delivery, management, case report

## Abstract

Uterine prolapse complicating pregnancy is extremely rare. However, in some cases, serious complications may appear such as miscarriage, preterm labor, infection, fetal demise, and maternal death. Management is essentially conservative but surgical intervention is always possible. We report a case of uterine prolapse in a young 24-year-old parturient, para 4, gravida 4 at 36 weeks of gestation. She was presented to the obstetric emergency, with uterine contraction and rupture of the amniotic sac. She successfully gave birth to a male baby weighing 2600 g by assisted vaginal delivery. We opted for conservative treatment and there were no fetal or maternal complications. A pessary was placed at the postpartum. Early diagnosis and good monitoring during pregnancy are essential to avoid complications; improve the prognosis of pregnancy and increase women´s chances to benefit from the advantages of conservative treatment.

## Introduction

Uterine prolapse is the descent of the uterus from its natural anatomical position, in the vagina. It is a common benign gynecologic condition, which is a part of a pathologic entity known as pelvic organ prolapse (POP). The ethnic and genetic differences of women around the world explain the lower prevalence of this pathology in African women who seem to have a stronger structure of the pelvic floor and supporting tissues; as well as; their good response to different traumas, compared to White and Hispanic women [1]. Uterine prolapse is usually the prerogative of elderly menopausal women, it occurs rarely in young women still in genital activity. Moreover, it remains uncommon during pregnancy with an estimated incidence of 1 in 10,000 to 15,000 deliveries [2], but more studies are necessary to evaluate the true incidence. Generally, POP is not life-threatening, but the occurrence of such a pathological entity during pregnancy is not without risk. It can be the cause of several complications during pregnancy, childbirth, or even postpartum, which can lead to fetal and maternal death [3]. Only a few cases have been reported in the literature and the management of which vary considerably. It ranges from a simple conservative treatment consisting of manual reduction of the prolapse with close monitoring, and installation of a pessary, to a more invasive laparoscopic intervention. Here, we present a case of uterine prolapse complicating a third-trimester pregnancy in a young, fourth-period woman with no particular pathological history. To our knowledge, it´s the first case notified in Morocco.

## Patient and observation

**Patient information:** this is a young rural parturient aged 24-years-old, 4 gravida, 4 para, with 3 living children. Her first child was born six years ago when she was 16-years-old. The three deliveries took place through the vagina, without any instrumental extraction, giving birth to eutrophic newborns. Moreover, the parturient had no particular pathological history related to pelvic traumas pelvic organ prolapse, or urinary or fecal incontinence. She reported the notion of sensation of pelvic heaviness with grade I cervical descent since the 16^th^ week of gestation, which she managed to reduce by herself.

**Timeline of current episode:** she was presented to the obstetric emergency, with uterine contraction and rupture of the amniotic sac, just one hour before her admission, following overload work.

**Figure 1:**
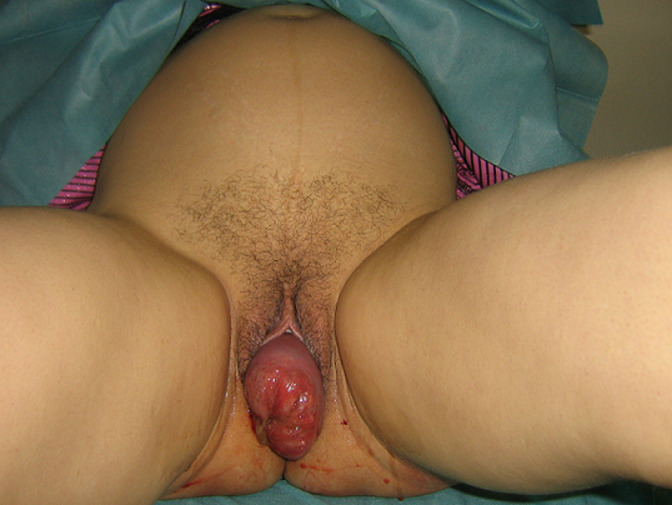
uterine prolapse at 36 weeks of pregnancy with rupture of the membranes and amniotic fluid leakage

**Clinical findings:** the pregnancy was estimated at 36 weeks of gestation, her clinical examination was unremarkable; the obstetrical examination noted a prolapse of the cervix with amniotic fluid, it was elongated; edematous with an ectropion and irreducible (Figure 1).

**Diagnostic assessment:** the parturient benefited from an obstetric ultrasound which objectified a normal-looking fetus with a biometry of 36-37 weeks of amenorrhea and a reduced amount of amniotic fluid. The recording of the fetal heart rate was normal and the infection assessment carried out was negative. The os was closed at her admission, but the evolution was marked by a fast labor onset. The delivery was uneventful vaginally, giving birth to a healthy male newborn, with a birth weight of 2600 g. The placenta and membranes were delivered spontaneously, without complications.

**Diagnosis:** the diagnosis of uterine prolapse was clinically easy and did not pose a problem.

**Therapeutic interventions:** the patient was put under careful monitoring in Trendelenbourg position and her prolapsed cervix was reduced. In the postpartum, a cervicovaginal smear was performed and was normal. A pessary was offered to the woman and put in place 4 days after delivery, then she was discharged on the same day and referred for physiotherapy care.

Follow-up and outcome of interventions: her follow-up for six months was good.

**Informed consent:** we obtained informed consent from the patient involved in this case report.

## Discussion

Pelvic organ prolapse (POP) is the herniation of the pelvic organs in the canal vagina due to dysfunction of pelvic floor muscles and supporting structures. It affects about 50% of women admitted for gynecological examination [4]. It negatively affects the daily life of women, especially their sexual activity, suffering from dyspareunia, and pelvic pain. It is well known that POP risk factors are multiparity, advancing age, vaginal delivery, obstructed labor, fetal macrosomia, obstetric traumas, obesity, congenital connective tissue disorders, racial and genetic factors, collagen abnormalities, gynecologic surgery, and a chronic increase in intra-abdominal pressure [4,5]. However, pregnancy and childbirth are thought to be the most involved risk factors for prolapse, and the most critical physiological events in reproductive female life, threatening seriously the woman´s pelvic floor integrity [5]. Uterine prolapse may precede or occur during pregnancy mostly in the third trimester, and in most cases, pre-existing uterine prolapse can resolve spontaneously during pregnancy. However, it can persist or recur after delivery [1]. It is a rare and serious disease; the main cause is Pelvic Floor Disorders (PFD) which lead to weakness of structures and tissues that support pelvic organs. Indeed, pregnancy induces physiological changes in the vaginal wall, in associated supporting tissues, and in the uterine cervix leading to its growth and softening; as well as hypertrophy and relaxation of the supporting ligaments. Steroid hormones change during pregnancy ensures that vaginal delivery takes place in good conditions [6]. In particular progesterone and the effect of relaxin may play also a role by causing growth and softening of the cervix could explain this descent of the pelvic organ prolapse, as well as multiparity and workload [7].

Perineal trauma during natural birth is often considered the main risk factor for pelvic floor disorders. However, this obstetric event cannot alone explain the occurrence of pelvic floor disorders since nearly 20% of women giving birth by cesarean section are also affected by these disorders [8]. This, therefore, suggests cesarean section is not entirely protective for pelvic floor damage, compared to vaginal delivery, and confirms the existence of a specific role of pregnancy, independently of the mode of delivery [3]. Pregnancy complications related to uterine prolapse include antepartum, peripartum, and postpartum complications. The main complications are abortion and preterm labor, cervical dystocia, prolonged or stagnation of labor; retention of urine and urinary tract infection, premature rupture of membranes, chorioamnionitis, uterine sepsis, fetal demise, and maternal death [9]. The management of gravid uterine prolapse should be considered according to its stage, the gestational age of pregnancy, the associated complications, and the patient´s choice. In addition to the clinical examination, POP may be evaluated and classified by translabial or transperineal ultrasound and magnetic resonance imaging. Any induction of labor, instrumental or non-instrumental maneuver must be avoided in this condition [4].

The treatment of uterine prolapse and its complications during pregnancy It must be taken case by case and vary from conservative treatment such as taxis of the prolapsed uterus which will protect the cervix from local trauma and prevent the possibility of incarceration; bed rest in the Trendelenburg position; intimate hygiene to avoid infection and the use of adequate vaginal pessary which should not be removed until the onset of labor [7], to minimally invasive surgery, when all of these conservative solutions fail or are impossible to applied. The most preferred delivery mode is a cesarean section near term to avoid complications during labor [3]. However, vaginal deliveries have been reported in the literature concerning non-externalized prolapse with labor occurring spontaneously before 37 weeks. In our case, the care of the parturient did not pose any obstetrical problem, and the placement of the pessary after childbirth was successful with good compliance. This case is reported to highlight the importance of good function of the pelvic floor and the role of POP prevention, especially among pregnant women since parity and delivery are the factors that have the biggest impact on developing POP in the future [5]. Even though POP is increased among older women, young women are not safe and should learn how to prevent pelvic-floor disorders and their complications during pregnancy and labor and how to rehabilitate pelvic floor muscles after labor. Moreover, pelvic floor muscle training seems to have in POP and PFD prevention and even treatment in the early stages of these conditions, without forgetting the important role of a healthy lifestyle: reduction of workload and avoidance of lifting heavy objects during pregnancy and postpartum.

## Conclusion

Despite its rarity, gravid uterine prolapse can be the cause of serious complications both maternal and fetal. Early diagnosis and good management are very important to avoid complications and ensure a safe pregnancy. It is associated with significant obstetric morbidity, its management depends on the gestational age, the stage of the prolapse, the associated complications, and the patient´s preferences. Conservative treatment is by far the most adopted and prophylactic cesarean section near term is the safest mode of delivery.
